# Polymyxin for treatment of ventilator-associated pneumonia in a setting of high carbapenem resistance

**DOI:** 10.1371/journal.pone.0237880

**Published:** 2020-08-19

**Authors:** Thalita Bento Talizin, Cláudia Maria Dantas de Maio Carrilho, Cintia Magalhães Carvalho Grion, Lucienne Tibery Queiroz Cardoso, Marcos Toshiyuki Tanita, Karine Maria Boll, Ivanil Aparecida Moro Kauss, Josiane Festti, Camila Ribeiro Lopes, Leticia Maria Alves da Silva, Isabella Patruceli de Azevedo, Késia Paes, Eduardo Alexandrino Medeiros

**Affiliations:** 1 Comissão de Epidemiologia Hospitalar, Escola Paulista de Medicina, Universidade Federal de São Paulo, São Paulo, São Paulo, Brazil; 2 Medical School, Universidade Estadual de Londrina, Londrina, Paraná, Brazil; 3 Hospital Universitário da Universidade Estadual de Londrina, Universidade Estadual de Londrina, Londrina, Paraná, Brazil; Vita Salute University of Milan, ITALY

## Abstract

**Objectives:**

To analyse the use of polymyxins for the treatment of ventilator-associated pneumonia (VAP) at a teaching hospital where carbapenem-resistant gram-negative bacteria are endemic.

**Patients and methods:**

This was a historical cohort study of patients receiving polymyxins to treat VAP in ICUs at a public university hospital in southern Brazil between January 1, 2017 and January 31, 2018.

**Results:**

During the study period, 179 cases of VAP were treated with polymyxins. Of the 179 patients, 158 (88.3%) were classified as having chronic critical illness. Death occurred in 145 cases (81.0%). Multivariate analysis showed that the factors independently associated with mortality were the presence of comorbidities (*P*<0.001) and the SOFA score of the day of polymyxin prescription (*P*<0.001). Being a burn patient was a protective factor for mortality (*P*<0.001). Analysis of the 14-day survival probability showed that mortality was higher among the patients who had sepsis or septic shock at the time of polymyxin prescription (*P* = 0.028 and *P*<0.001, respectively). *Acinetobacter baumannii* was identified as the etiological agent of VAP in 121 cases (67.6%). In our cohort, polymyxin consumption and the incidence density of VAP were quite high.

**Conclusions:**

In our study, comprised primarily of chronically critically ill patients, there was a high prevalence of VAP caused by multidrug-resistant bacteria, consistent with healthcare-associated infections in low- and middle-income countries. Presence of comorbidities and the SOFA score at the time of polymyxin prescription were predictors of mortality in this cohort. Despite aggressive antimicrobial treatment, mortality was high, stressing the need for antibiotic stewardship.

## Introduction

Healthcare-associated infections (HAI) are a public policy concern in low- and middle-income countries, particularly in terms of providing technological support and qualified human resources, as well as improving antimicrobial stewardship programs and reducing the rates of microbial resistance [1]. Studies have shown that, in Brazil, the prevalence of HAIs in hospitals is 10.8% and pneumonia is the most common HAI [2]. In ICUs in Brazil, the prevalence of HAIs is considerably higher (51.2%) and those caused by *Enterobacteriaceae* or non-fermenting bacteria are quite common [3]. In the ICU, patients undergo mechanical ventilation, which can lead to ventilator-associated pneumonia (VAP). Multidrug-resistant *Acinetobacter baumannii* is one of the most common etiologic agents of VAP in critically ill patients, which continues to be a major therapeutic challenge [4].

In recent years, polymyxins once again have become one of the only effective drug classes for treating infection with multidrug-resistant gram-negative bacteria such as *A*. *baumannii*, *Pseudomonas aeruginosa* and *Enterobacteriaceae*. Polymyxins are toxic, and there are no data regarding the best therapeutic dose or antimicrobial combination to provide efficacy. There have been only a few studies analysing the profiles of polymyxin users and attempting to determine the best dosage [5,6].

In many hospitals in Brazil, especially in tertiary care hospitals, the local microbiota profile warrants the empirical prescription of polymyxins, given that carbapenem-resistant microorganisms are endemic in some parts of the country. The large-scale empirical use of this drug class is worrisome, not only because polymyxins are toxic but also because it creates antimicrobial selection pressure. There have been few epidemiological studies of the use of polymyxins. Although mortality is high among ICU patients treated with polymyxins, it is difficult to establish a single cause of death in such patients, due to the concomitance of events [7].

In view of the history and current profile of polymyxin use, we developed this study to evaluate the profile and clinical evolution of patients treated with drugs in this class, comparing survivors and non-survivors. The aim was to describe the use of polymyxins in the treatment of VAP at a teaching hospital in Brazil, where carbapenem-resistant gram-negative bacteria are endemic.

## Patients and methods

### Setting

This study was conducted at the *Hospital Universitário da Universidade Estadual de Londrina* (HU-UEL, University Hospital of the Londrina State University), in the city of Londrina, Brazil. The HU-UEL is a public referral hospital, providing comprehensive care via the Brazilian Unified Health Care System, and has approximately 320 beds. The adult ICU of the HU-UEL has 20 beds for clinical and surgical patients >14 years of age, with a 90.51% average occupancy rate. The HU-UEL Burn Treatment Centre has a specialised ICU with six beds and an average occupancy rate of 91.96%.

### Ethics

The research protocol was developed in accordance with Brazilian National Health Council Resolution no. 466/2012, which deals with the ethical aspects of research involving human beings in Brazil. The study was approved by the Research Ethics Committee of the *Universidade Federal de São Paulo* (Ruling no. 3564733; Reference no. 95308418.5.0000.5505). The study had a retrospective design and the Ethics Committee waived the requirement for informed consent. To perform the analysis, all data was on anonymously.

### Study design and patients

This was a historical cohort study. The cohort comprised patients receiving polymyxin, either empirically or on the basis of culture results, to treat VAP during hospitalisation in the HU-UEL adult ICU or Burn Treatment Centre ICU, between January 1, 2017 and January 31, 2018. We selected consecutive patients. The patients treated with polymyxin were identified by consulting the drug dispensing database of the HU-UEL Pharmacy. We initially selected all ICU patients receiving polymyxin during the study period and subsequently excluded those in whom polymyxin was prescribed for conditions other than VAP.

### Definitions

We defined VAP on the basis of the CDC criteria, which is a flowchart of clinical, microbiological, serological and anatomopathological criteria to establish diagnosis of this condition [8]. Sepsis and septic shock were defined according to the diagnostic criteria established by The Third International Consensus Definitions for Sepsis and Septic Shock. Sepsis is defined as organ dysfunction caused by an infection, quantified by an increase of ≥ 2 points in the SOFA score. Septic shock is defined as the need for a vasopressor to maintain mean arterial pressure ≥ 65 mmHg and persistence of lactate ≥ 2 mmol/L despite volume resuscitation [9]. Chronically critically ill patients were identified as described in the Chronically Critically Ill Population Payment Recommendations report, which classifies patients with one or more of the following criteria: need for prolonged mechanical ventilation, tracheostomy, organ failure of ≥ 2 systems, sepsis, wounds and debilitating factors [10].

### Patient selection criteria

The study included patients in whom polymyxin was used to treat VAP in the adult ICU or Burn Treatment Centre ICU of the HU-UEL. Patients under 18 years of age were excluded, as were those treated with polymyxin for less than 48 h and those for whom medical records were incomplete or missing.

### Study variables

We collected data related to the following: demographic characteristics—age and gender; clinical characteristics—comorbidities, the diagnosis at hospital admission and the reason for ICU admission; VAP—the diagnosis of sepsis or septic shock and the lactate level at the time of polymyxin prescription; the prescription of polymyxin—the type of polymyxin prescribed, the duration of treatment, the length of hospital stay before polymyxin prescription, where the polymyxin was prescribed (in the ward or in the ICU), the type of therapy (empirical or based on culture results) and the treatment regimen (the drug or drugs administered); microbiology—the microorganism isolated and the antimicrobial susceptibility test results; clinical interventions—hemodialysis and vasoactive drug use; and hospitalisation—the length of the ICU stay, readmission to the ICU, the length of hospital stay and the outcome. At admission, the APACHE II score and the Simplified Acute Physiology Score 3 (SAPS 3) were calculated as prognostic scores. Also at admission, as well as on the first day of polymyxin use, the SOFA and Simplified Therapeutic Intervention Scoring System scores were calculated as scores for organ dysfunction and nurse workload respectively.

### Microbiology procedures

All microbiology procedures were performed in samples of tracheal aspirate. Samples were collected from patients with a patent airway by means of vacuum aspiration via a sterile tracheal aspiration tube. The material was aspirated directly into a polystyrene collection bottle, sterilised with ethylene oxide.

Tracheal aspirate samples were processed, and those in which there was growth of ≥10^6^ cfu/mL of infectious process-related microorganism colonies were identified in an automated system (VITEK 2; bioMérieux, Marcy I’Étoile, France). Antimicrobial susceptibility testing was performed following the guidelines established by the CLSI. The breakpoints used for all antimicrobials were those established in CLSI document M100 in 2017 [11]. The polymyxin breakpoints for *Enterobacteriaceae* were established according to the 2017 EUCAST guidelines [12]. For all samples, carbapenem sensitivity was determined by disk diffusion, in accordance with the CLSI criteria for each microorganism. All bacterial strains were tested for polymyxin susceptibility by epsilometer test (Etest; bioMérieux) or by microdilution, in accordance with the CLSI criteria.

### Polymyxin administration

The administration of the antimicrobials followed recommendations in the literature regarding the doses and adjustments for polymyxin B [13,14] and colistin [15].

### Statistical analysis

The normality of continuous variables was determined by the Kolmogorov–Smirnov test; those with a parametric distribution are expressed as mean and standard deviation, whereas those with a non-parametric distribution are expressed or as median and interquartile range (IQR). Student’s *t*-tests were used in order to compare the means of continuous variables with normal distribution and homogeneity of variance. The Mann–Whitney test was applied in order to compare non-parametric data. Categorical variables are expressed as absolute and relative frequencies, and the data were compared by using the chi-square test.

We performed logistic regression analysis to estimate risk factors for mortality, using the enter method for univariate analysis and the stepwise method for multivariate analysis. Variables entered the model at *P*<0.05 and exited at *P*>0.1. The effect of each variable was expressed as an odds ratio and a corresponding 95% confidence interval. Additionally a logistic regression was performed with the following variables: “age” and “burn injury”.

In-hospital mortality is described as frequency, and Kaplan–Meier survival curve analysis was performed. We calculated the incidence density of VAP ([number of cases of VAP in the ICU ∕ number of patients on mechanical ventilation per day] × 1000) and the consumption of polymyxins in defined daily dose (DDD) per 1000 patient-days. We also performed simple linear regression, calculating the coefficient of determination (*R*^*2*^) to evaluate the time series trend.

The risk of a type I error was set at <5%. Statistical analyses were performed with MedCalc Statistical Software, version 18.9 (MedCalc Software, Ostend, Belgium).

## Results

During the study period, polymyxins were prescribed for the treatment of HAI in 291 patients. Of those 291 patients, 46 were initially excluded: six were under 18 years of age; 39 received the antimicrobial for less than 48 h; and one was lost to follow-up. Another 66 patients were excluded because the polymyxin was prescribed for the treatment of a condition other than VAP. Therefore, the final sample comprised 179 patients. There were 15 patients who received two rounds of treatment and four who received three rounds, resulting in a total of 202 prescriptions. However, for the purposes of this study, we analysed only the first round of treatment in each patient.

As shown in Table 1, the median age of the patients receiving a polymyxin for the treatment of VAP in the ICU was 57 years (IQR: 40.0–70.75 years), and 80 patients (44.7%) were over 60 years of age. Men accounted for 117 (65.4%) of the 179 hospitalisations. Of the 179 patients, 130 (72.6%) had comorbidities, the most common being circulatory diseases, which were present in 76 (42.4%), followed by mental illness in 17 (13.1%), nervous system disorders in ten (7.7%), and neoplasms in seven (5.4%). The most common diagnosis at admission was a neurological disorder in 70 cases (39.1%), and the reason for hospitalisation was mainly clinical (i.e. not a surgical admission) in 124 (69.3%). The mean APACHE II score was 21.2 ± 7.1. The median length of ICU stay was 19 days (IQR: 12.0–29.0 days), and death in the ICU occurred in 116 cases (64.8%). The median length of hospital stay was 33 days (IQR: 19.0–52.0 days), and death during hospitalization occurred in 145 cases (81%). The 30-day hospital outcome analysis showed that death occurred in 77 cases (43%).

**Table 1:** Characteristics of patients receiving polymyxin for the treatment of VAP in the ICU, by outcome, at the *Hospital Universitário da Universidade Estadual de Londrina*, in the city of Londrina, Brazil, between January 1, 2017 and January 31, 2018.

Characteristic	All patients (*n* = 179)	Survivors (*n* = 34)	Nonsurvivors (*n* = 145)	*P*
Age (years), median (IQR)	57.0 (40.0–70.75)	36.5 (24.0–50.0)	60.0 (49.0–73.0)	< 0.001^a^
Male, *n* (%)	117 (65.4)	26 (76.5)	91 (62.8)	0.1315^b^
Comorbidities, *n* (%)	130 (72.6)	13 (38.2)	117 (80.7)	< 0.001^b^
Diagnosis at admission, *n* (%)				
neurological disorder	70 (39.1)	9 (26.5)	61 (42.1)	0.0944^b^
sepsis	42 (23.5)	6 (17.6)	36 (24.8)	0.3752^b^
burn	27 (15.1)	13 (38.2)	14 (9.7)	< 0.001^b^
other (surgical patients)	32 (17.9)	6 (17.6)	26 (17.6)	0.9691^b^
Reason for admission, *n* (%)				
clinical diagnosis	124 (69.3)	25 (73.5)	99 (68.3)	0.5512^b^
postoperative care after elective surgery	11 (20.0)	1 (3.0)	10 (6.9)	0.3887^b^
postoperative care after urgent surgery	44 (80.0)	8 (23.5)	36 (24.9)	0.8746^b^
APACHE II score, mean ± SD	21.2 ± 7.1	18.79 ± 6.34	21.8 ± 7.2	0.0265^c^
SAPS 3, mean ± SD	68.5 ± 16.7	59.35 ± 16.25	70.7 ± 16.0	< 0.001^c^
SOFA score, median (IQR)				
at admission	7.0 (5.25–9.75)	7.0 (5.0–8.0)	8.0 (5.75–10.0)	0.2620^a^
at polymyxin prescription	7.0 (6.0–10.0)	7.0 (5.0–8.0)	8.0 (6.0–11.0)	0.0093^a^
Simplified TISS 28 score, median (IQR)				
at admission	29.0 (26.0–33.0)	29.0 (26–34)	29.0 (26–32)	0.5511^a^
at polymyxin prescription	29.0 (26.0–33.0)	29.0 (26–32)	29.0 (26–33)	0.6980^a^
ICU stay (days), median (IQR)	19.0 (12.0–29.0)	22.0 (18.0–42.0)	18.0 (11.0–28.25)	0.0244^a^
ICU readmission, *n* (%)	39 (21.8)	7 (20.6)	32 (22.1)	0.8511^b^
Hospital stay (days), median (IQR)	33.0 (19.0–52.0)	47.5 (35.0–63.0)	28.0 (17.0–47.0)	< 0.001^a^
Treatment duration (days), median (IQR)	11.0 (5.0–14.75)	14.0 (14.0–18.0)	10.0 (5.0–14.0)	< 0.001^a^
Polymyxin prescription in the ICU, *n* (%)	157 (87.7)	31 (91.2)	126 (86.9)	0.4951^b^
Days from admission to polymyxin prescription, median (IQR)	12.0 (9.0–19.0)	10.0 (8.0–13.0)	13.0 (9.75–21.0)	0.0101^a^
Sepsis at polymyxin prescription, *n* (%)	123 (68.7)	19 (55.9)	104 (71.7)	0.0738^b^
Septic shock at polymyxin prescription, *n* (%)	37 (20.7)	4 (11.8)	33 (22.8)	0.1554^b^
Lactate (mmol/L), median (IQR)	1.7 (1.3–2.2)	1.4 (1.2–1.8)	1.8 (1.4–2.3)	0.0072^a^
Hemodialysis, *n* (%)	100 (55.9)	13 (38.2)	87 (60.0)	0.0218^b^
Vasoactive drug use, *n* (%)	168 (93.9)	29 (85.3)	139 (95.9)	0.0213^b^
Etiological agent, *n* (%)				
*Acinetobacter baumannii*	121 (67.6)	28 (82.4)	93 (64.1)	0.0417^b^
*Pseudomonas aeruginosa*	20 (11.2)	0 (0)	20 (13.8)	0.0219^b^
*Klebsiella pneumoniae*	16 (8.9)	4 (11.8)	12 (8.3)	0.5222^b^
Drug resistance, n (%)				
Carbapenem	159 (88.8)	30 (88.2)	129 (89.0)	0.9035^b^
Polymyxin	9 (5.0)	0 (0)	9 (6.2)	0.1371^b^
Aminoglycoside	98 (54.7)	19 (55.8)	79 (54.5)	0.8830^b^

VAP, ventilator-associated pneumonia; IQR, interquartile range; SD, standard deviation; SAPS 3, Simplified Acute Physiology Score 3; TISS, Therapeutic Intervention Scoring System.

^a^Mann–Whitney test.

^b^chi-square test.

^c^Student’s *t*-test.

Comparing survivors and non-survivors, we found that the median age was lower in the former (36.5 years versus 60.0 years; *P*<0.001). The proportion of patients with comorbidities was higher among the non-survivors (80.7% versus 38.2%; *P*<0.001). There were also differences between survivors and non-survivors in terms of the mean APACHE II score (18.8 versus 21.8; *P* = 0.0265), the mean SAPS 3 (59.35 versus 70.7; *P*<0.001) and the median SOFA score on the day of polymyxin prescription (7.0 versus 8.0; *P* = 0.0093). The median lactate level was higher among the non-survivors (1.8 mmol/L versus 1.4 mmol/L; *P* = 0.0072). Of the 179 patients, 158 (88.3%) were classified as having chronic critical illness.

Univariate analysis showed that the following risk factors were independently associated with in-hospital mortality: being ≥ 60 years of age (*P* = 0.001); having at least one comorbidity (*P*<0.001); the APACHE II score (*P* = 0.0288); the SAPS 3 (*P*<0.001); the SOFA score on the day of polymyxin prescription (*P* = 0.0073); and requiring hemodialysis (*P* = 0.0238). Being a burn patient was found to be a protective factor (*P*<0.001). In the multivariate analysis (Table 2), the presence of comorbidities retained its significance as a risk factor (*P*<0.001), as did the SOFA score on the day of polymyxin prescription (*P* = 0.0348), and being a burn patient retained its significance as a protective factor (*P* = 0.0051).

**Table 2:** Univariate and multivariate analyses to identify factors independently associated with in-hospital mortality in ICU patients receiving polymyxin for the treatment of VAP at the *Hospital Universitário da Universidade Estadual de Londrina*, in the city of Londrina, Brazil, between January 1, 2017 and January 31, 2018.

Factor	Univariate analysis			Multivariate analysis		
	OR	95% CI	*P*	OR	95% CI	*P*
18–59 years of age	0.205	0.080–0.526	0.001			
≥ 60 years of age	4.863	1.900–12.450	0.001			
At least one comorbidity	6.750	3.017–15.101	< 0.001	4.630	1.969–10.888	< 0.001
ICU readmission	1.092	0.435–2.739	0.8507			
Empirical prescription of polymyxin	1.697	0.738–3.902	0.2128			
Sepsis	2.002	0.929–4.313	0.0761			
Septic shock	2.209	0.726–6.726	0.1627			
Lactate > 2 mmol/L	2.314	0.943–5.676	0.0668			
APACHE II score	1.066	1.006–1.129	0.0288			
SAPS 3	1.045	1.019–1.072	< 0.001			
SOFA score at admission	1.082	0.950–1.233	0.2346			
SOFA score at polymyxin prescription	1.214	1.053–1.399	0.0073	1.190	1.012–1.400	0.0348
Hemodialysis	2.423	1.124–5.219	0.0238			
Burn patient	0.172	0.071–0.418	< 0.001	0.244	0.091–0.654	0.0051

VAP, ventilator-associated pneumonia; SAPS 3, Simplified Acute Physiology Score 3; TISS, Therapeutic Intervention Scoring System.

Logistic regression analysis showed that age and being a burn patient were independently statistically significant (P<0.001). Odds ratios calculated from this adjusted model indicated that mortality was higher in older patients (OR, 1.057; 95% CI, 1.031–1.083), while mortality was lower in burn patients (OR, 0.258; 95% CI, 0.094–0.709).

**Fig 1:**
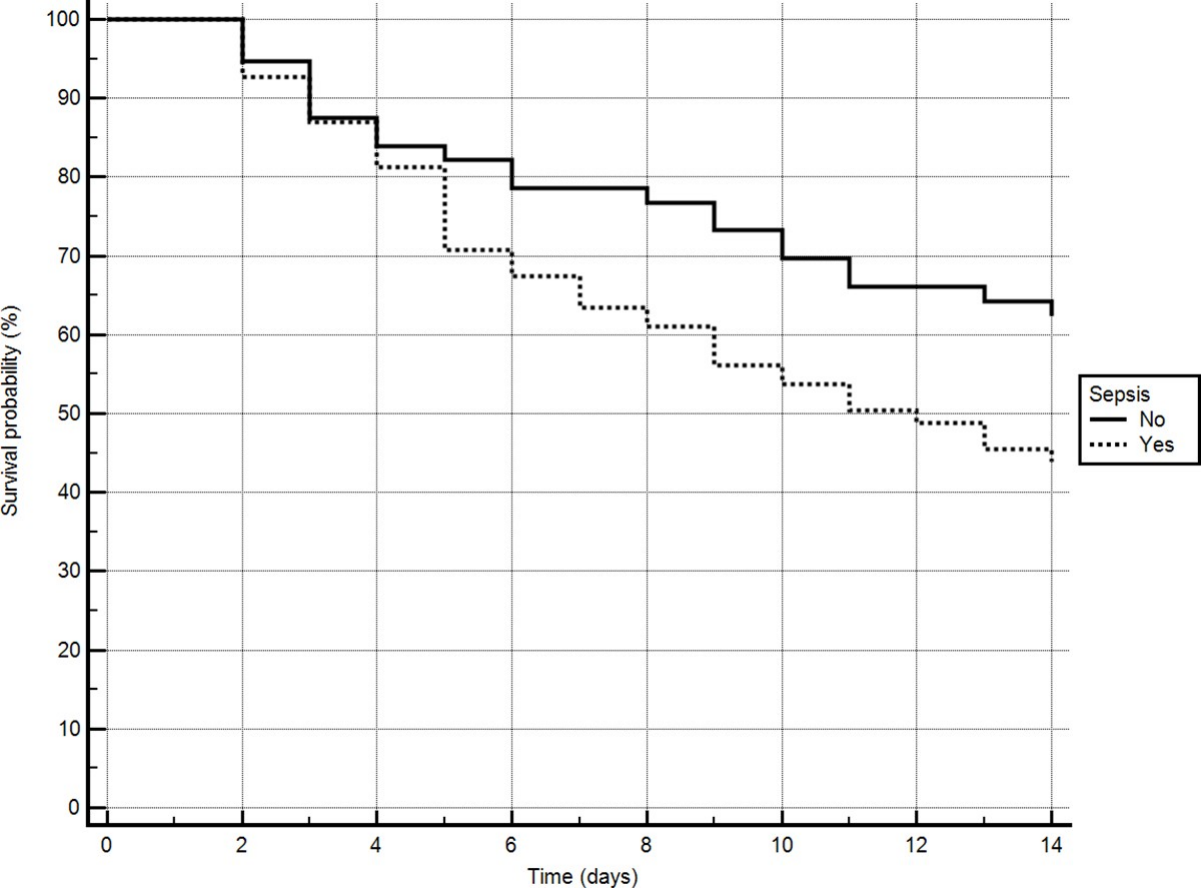
Analysis of the probability of 14-day survival in ICU patients with and without sepsis on the first day of polymyxin administration to treat ventilator-associated pneumonia at the *Hospital Universitário da Universidade Estadual de Londrina*, in the city of Londrina, Brazil, between January 1, 2017 and January 31, 2018. Log-rank test, *P* = 0.0284.

Analysis of the 14-day survival probability showed higher mortality for patients with sepsis at the time of polymyxin prescription (log-rank test, *P* = 0.028), as shown in Fig 1.

**Fig 2:**
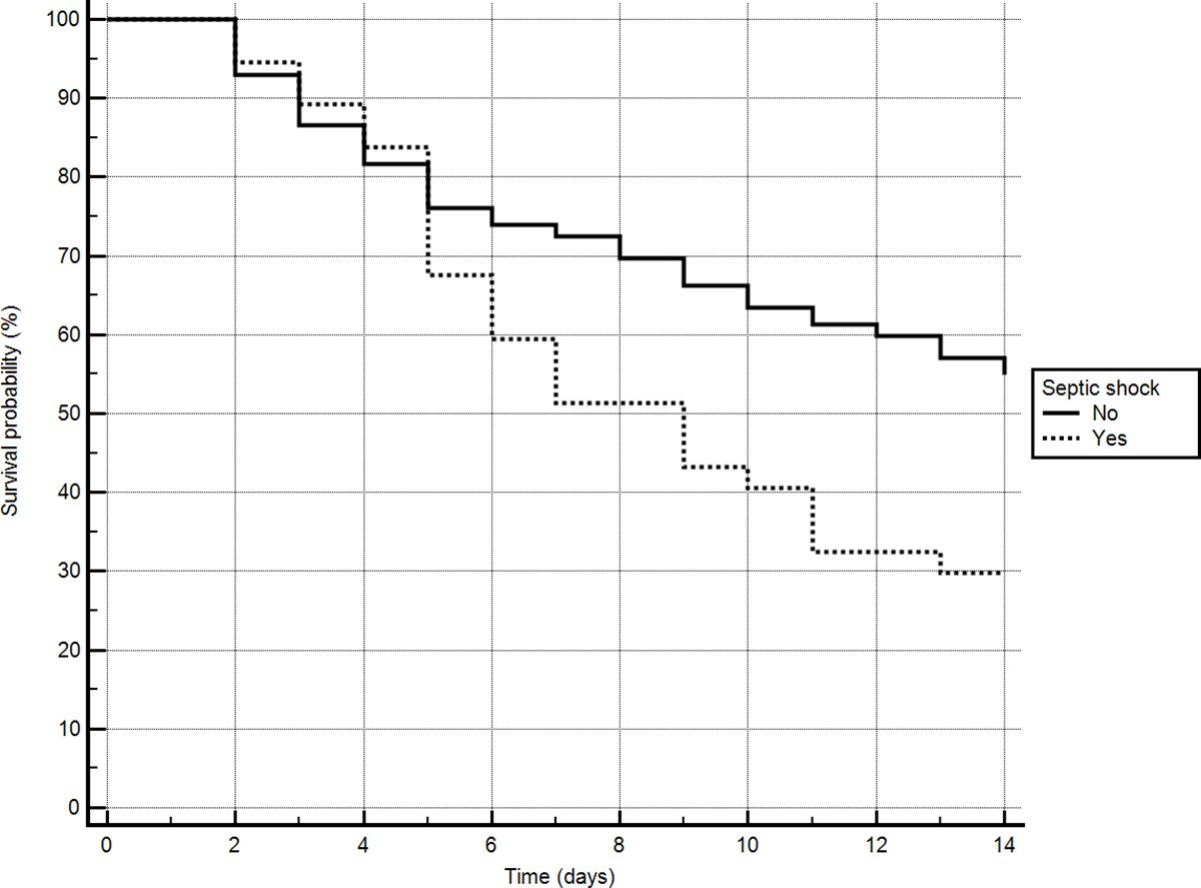
Analysis of the probability of 14-day survival in ICU patients with and without septic shock on the first day of polymyxin administration to treat ventilator-associated pneumonia at the *Hospital Universitário da Universidade Estadual de Londrina*, in the city of Londrina, Brazil, between January 1, 2017 and January 31, 2018. Log-rank test, *P* = 0.0065.

Mortality at 14 days was also higher among the patients with septic shock at the time of polymyxin prescription (log-rank test, *P* = 0.006), as shown in Fig 2.

The polymyxin therapy was empirical in 64 cases (35.8%): 47 patients (73.4%) had a carbapenem resistant bacteria and 17 (26.6%) had no microbiological confirmation. Comparing the groups of patients receiving polymyxin based on culture results and those receiving it as empirical therapy, we found no differences in terms of mortality (78.3% versus 85.9%; *P* = 0.21), the mean SAPS 3 (67.7 versus 70.0; *P* = 0.38) or the median SOFA score on the day of polymyxin prescription (7 versus 8, *P* = 0.27).

The microorganism responsible for VAP was *A*. *baumannii* in 121 cases (67.6%), *P*. *aeruginosa* in 20 cases (11.2%) and *Klebsiella pneumoniae* in 16 cases (8.9%). The antimicrobial used most commonly during the study period was polymyxin B, whereas polymyxin E was used in only five patients (2.8%). Antimicrobials were prescribed in the ward (i.e., before ICU admission) in 22 cases (12.3%). A polymyxin was administered as monotherapy in 14 cases (7.8%). The drugs most commonly used in combination with a polymyxin were carbapenems in 85 cases (51.5%); tigecycline in 53 cases (29.6%) and aminoglycosides in 12 cases (6.7%). Regarding the outcome of hospitalisation, there were no differences between monotherapy and any of the combination therapy regimens (*P* = 0.640).

For *A*. *baumannii*, resistance to carbapenems was observed in 122 isolates (97.6%) and resistance to polymyxins was observed in seven (5.6%). Among the isolates of *P*. *aeruginosa*, there was resistance to carbapenems in 23 (92.0%) and no resistance to polymyxins. Among *K*. *pneumoniae* isolates, carbapenem resistance was observed in 18 (94.7%) and polymyxin resistance was observed in two (10.5%). All of the patients infected with polymyxin-resistant microorganisms died.

**Fig 3:**
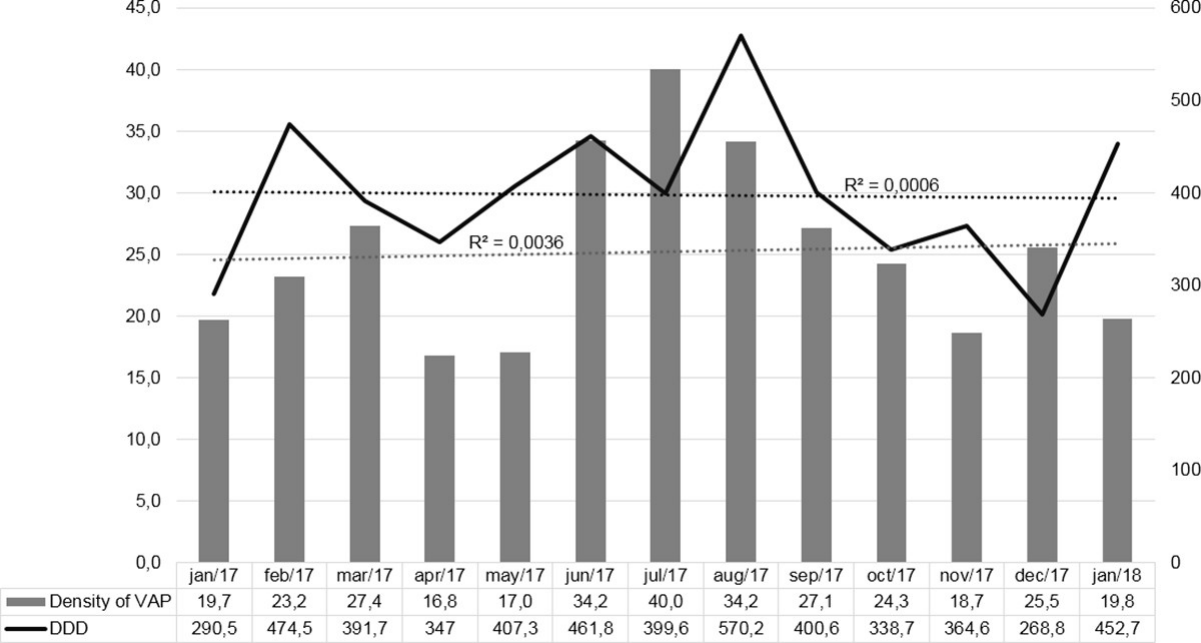
Time series for the distribution of the incidence density of Ventilator-Associated Pneumonia (VAP) and polymyxin consumption, at a Defined Daily Dose (DDD) per 1000 patient-days, in ICUs at the *Hospital Universitário da Universidade Estadual de Londrina*, in the city of Londrina, Brazil, between January 1, 2017 and January 31, 2018. VAP, ventilator-associated pneumonia; DDD, defined daily dose.

Polymyxin consumption in both ICUs was quantified in DDD per 1000 patient-days. The monthly distribution of the incidence density of VAP is shown in Fig 3. Simple linear regression showed no trend in the number of VAP cases (*R*^*2*^ = 0.0034) or polymyxin consumption (*R*^*2*^ = 0.0006).

## Discussion

Our cohort was composed primarily of chronically critically ill patients, as evidenced by the severity scores and the length of the hospital stays. The 30-day mortality rate in our cohort was higher than the 29.41% reported in another study of critically ill patients treated with polymyxin in Brazil [16], although our patients had higher APACHE II scores. In that study, the respiratory tract was also the most common site of infection treated with a polymyxin.

In recent decades, there has been growing interest in the study of chronically critically ill patients who survive the initial acute insult but cannot be weaned from life support, therefore requiring prolonged hospitalisation, which increases the demand for resources. Such patients develop immune exhaustion due to the combination of invasive procedures and morbidity, promoting prolonged hospitalisation and a higher incidence of HAI [17,18].

In-hospital mortality was high in our cohort, and the variables independently associated with mortality were the presence of a comorbidity and severe organ dysfunction. An unexpected finding was that being a burn patient was a protective factor for mortality. One plausible explanation is that most burn patients are younger and typically have no comorbidities, given that the majority of the burn cases treated at the HU-UEL Burn Treatment Centre are the result of occupational accidents [19]. This hypothesis could be confirmed by logistic regression, which demonstrated that burn injury remained as an independent protective factor after adjusting for age. Approximately half of the in-hospital deaths occurred during the first 30 days after admission, which indicates that some of the patients survived the acute episode but remained hospitalised for a long period, eventually succumbing during the hospital stay. For these reasons and because of the complexity of the critically ill cases, it is unlikely that mortality can be attributed solely to infection.

Choosing the proper empirical therapy is a challenge for the intensive care practitioner, as local epidemiology, host risk factors and follow-up culture should be considered in order to adapt the spectrum of the antimicrobial therapy on a case-by-case basis. The use of polymyxins to treat VAP should be reserved for sites where the prevalence of multidrug-resistant bacteria is high and there is experience in the use of this class of drug, according to the Infectious Diseases Society of America/American Thoracic Society guidelines [20]. There are still knowledge gaps regarding the use of polymyxins, especially in relation to their toxicity, pharmacokinetics and pharmacodynamics [5].

Because of the local epidemiology, monotherapy was used in only 7.8% of the cases evaluated in our study, and it was not possible to draw conclusions on the basis of the results of comparisons between monotherapy and combination therapy. There is no consensus in the literature as to the best polymyxin regimen for each bacterial species, and the exact molecular mechanisms of combination therapy are not known. The supposition that combination therapy is superior needs to be tested in controlled studies [21,22].

In our cohort, there was a predominance of infections with *A*. *baumannii*, which is one of the pathogens most often isolated from ICU patients. The risk factors for infection with *A*. *baumannii*, which have been described in the literature [23,24], include severe disease at admission, previous colonisation, prolonged ICU stay, advanced age, and previous use of carbapenem or third-generation cephalosporins. Worldwide, the resistance of *A*. *baumannii* to polymyxins is underestimated by the methodologies adopted to test sensitivity. There can be heteroresistance in isolates classified as susceptible. Antimicrobial resistance among non-fermenting bacteria, especially *A*. *baumannii* resistance to carbapenems, has been increasing in recent years, leading to more widespread use of polymyxins to treat this condition [23]. Another study of *A*. *baumannii* pneumonia in critically ill patients in Brazil found that the SOFA score at the time of diagnosis is a risk factor for mortality [24], as was observed in our study.

In the present study, 14-day mortality was higher among the patients who were diagnosed with sepsis or septic shock than among those who were not. The Sepsis PREvalence Assessment Database study, conducted in ICUs throughout Brazil, estimated the incidence density of sepsis to be 36.3 cases per 1000 patient-days and the rate of sepsis-related mortality to be 55.7%. In that study, limited availability of resources was independently associated with higher mortality [25]. In Brazil, the incidence of sepsis and the associated in-hospital mortality rates have increased in recent years, sepsis-related mortality being higher at public hospitals than at private hospitals [26].

Other low- and middle-income countries have similar problems with multidrug resistance. In Asia, *A*. *baumannii* is one of the leading causes of carbapenem-resistant HAI, especially in the ICU [27]. A study conducted at a tertiary care hospital in South Korea showed prescription of drugs used in the treatment of infections with multidrug-resistant bacteria, including carbapenems, glycopeptides, oxazolidinones, polymyxins and glycylcyclines, showed a trend toward an increase between 2004 to 2013. The authors found that the DDD per 1000 patient-days in the ICU was 82.11 for carbapenem and 32.88 for polymyxin [28]. Worldwide, there was an increase in the prescription of last-resort antimicrobials, such as polymyxins, from 2000 to 2015 [29]. The consumption of polymyxins in our study was much higher than that reported at other facilities. Our study pooled the admissions to both of the ICUs in our hospital, which has no intermediate care unit. Therefore, chronically critically ill patients are assigned to specialised intensive care beds, which affects long-term hospitalisation indicators and the average occupancy rate.

In the present study, we have addressed scenarios that are increasingly prevalent in low- and middle-income countries: multidrug-resistant bacterial infections; restricted treatment options; critically ill patients; and prolonged hospitalisation. Further epidemiological studies are warranted in order to increase understanding of those scenarios and to identify risk factors for VAP requiring polymyxin.

Our study has certain limitations, including those inherent to a historical cohort design. We evaluated a sample of consecutive patients at a hospital where carbapenem-resistant microorganisms are endemic, and caution should therefore be exercised when generalising these results to other populations. Future studies should involve microbiological evaluation of isolates with clonal analysis, ideally comparing patients exposed and not exposed to polymyxins, to assess mortality and toxicity as outcomes. There is a need for a broader discussion regarding the use of broad-spectrum antimicrobial agents for the treatment of infections in patients hospitalised for prolonged periods.

We conclude that the presence of comorbidities and the SOFA score on the day of polymyxin prescription retained its significance as a risk factor for mortality and being a burn patient retained its significance as a protective factor. Analysis of the 14-day survival probability showed higher mortality for patients with sepsis and with septic shock at the time of polymyxin prescription. The microorganism responsible for VAP was *A*. *baumannii* in most cases. Regarding the outcome of hospitalisation, there were no differences between monotherapy and any of the combination therapy regimens. Carbapenem resistance and polymyxin consumption were high.
